# The Role of miR-31-5p in the Development of Intervertebral Disc Degeneration and Its Therapeutic Potential

**DOI:** 10.3389/fcell.2021.633974

**Published:** 2021-03-18

**Authors:** Yong Zhou, Mingsi Deng, Jiqing Su, Wei Zhang, Dongbiao Liu, Zhengguang Wang

**Affiliations:** ^1^Department of Orthopaedics, The Third Xiangya Hospital of Central South University, Changsha, China; ^2^Department of Stomatology, Changsha Stomatological Hospital, Changsha, China; ^3^Department of Oncology, Changsha Central Hospital Affiliated to Nanhua University, Changsha, China; ^4^Institute of Clinical Pharmacology, Central South University, Changsha, China

**Keywords:** IDD, miR-31-5p, SDF-1/CXCR7, Col II, therapeutic potential

## Abstract

Intervertebral disc degeneration (IDD) refers to the abnormal response of cell-mediated progressive structural failure. In order to understand the molecular mechanism of the maintenance and destruction of the intervertebral disc, new IDD treatment methods are developed. Here, we first analyzed the key regulators of IDD through microRNAs microarrays. Then, the level of miR-31-5p was evaluated by qRT-PCR. The association between miR-31-5p and Stromal cell-derived factor 1 (SDF-1)/CXCR7 axis was assessed by 3′-untranslated region (UTR) cloning and luciferase assay. The apoptosis of cells under different treatments was evaluated by flow cytometer. The cell proliferation was assessed by EdU assay. After IDD model establishment, the discs of mice tail were harvested for histological and radiographic evaluation in each group. Finally, the protein levels of SDF-1, CXCR7, ADAMTS-5, Col II, Aggrecan, and MMP13 were assessed by western blot. The results show that miR-31-5p is a key regulator of IDD and its level is down-regulated in IDD. Overexpression of miR-31-5p facilitates nucleus pulposus cell proliferation, inhibits apoptosis, facilitates ECM formation, and inhibits the level of matrix degrading enzymes in NP cells. The SDF-1/CXCR7 axis is the direct target of miR-31-5p. miR-31-5p acts on IDD by regulating SDF-1/CXCR7. *In vitro* experiments further verified that the up-regulation of miR-31-5p prevented the development of IDD. In conclusion, overexpression of miR-31-5p can inhibit IDD by regulating SDF-1/CXCR7.

## Introduction

Epidemiological statistics show that with the development of economy and society, the prevalence of low back pain worldwide has been increasing year by year, which has surpassed spinal cord injury and become one of the diseases with the highest disability rate (Murray and Lopez, 2013). The main root cause of low back pain is intervertebral disc degeneration (IDD) (Richardson et al., 2016). The intervertebral disc consists of a central, gelatinous nucleus pulposus surrounded by annulus fibrosis. The occurrence of degeneration will lead to the imbalance of the internal environment of the intervertebral disc, loss of tissue hydration, inflammation, and loss of extracellular matrix, which will lead to a decrease in the height of the intervertebral disc, destruction of the annulus fibrosus structure, and a gradual loss of normal physiological structure and function (Ghannam et al., 2017). IDD is a chronic process of degradation and destruction of extracellular matrix protein (ECM). The degradation products of ECM may trigger or further promote the inflammatory response interrelated to IDD and low back pain (Nies et al., 1991). Therefore, inhibiting the phenotypic abnormality of nucleus pulposus (NP) cells is the crucial to preventing the progression of IDD.

The pathogenesis of IDD is interrelated to many biological and genetic regulators (Tan et al., 2018; Cui and Zhang, 2020), but it has recently been revealed that microRNAs (miRNAs) are important regulators of the development of IDD. miRNAs regulate protein level by binding to the 3′-untranslated region of mRNAs, and then play a part in a variety of physiological and pathological processes (Bartel, 2004). miRNAs are key regulators of a variety of cellular physiological processes, including proliferation, differentiation, apoptosis, survival, and morphogenesis. A large number of studies have manifested that miRNAs are widely present in tissues and organs, and play a regulatory part in the occurrence and development of various diseases (Hammond, 2015). Abnormal miRNA level is present in various musculoskeletal diseases, such as osteoporosis and rheumatoid arthritis (Nakasa et al., 2008; Lei et al., 2011; Kolhe et al., 2017). More and more evidence support that miRNAs play a part in the process of causing IDD (Song et al., 2013; Ji et al., 2016a,b). The study found that miR-494, miR-27a, miR-155, miR-93, miR-146a, miR-377, miR-100, miR-21, miR-10, miR-146a, and other miRNA s have been reported to be interrelated to the development of IDD (Wang et al., 2015). However, the association between miR-31-5p and IDD is still less studied.

miR-31-5p is one of the most popular candidate genes (Yu et al., 2018). Emerging evidence shows that miR-31-5p is an oncogenic or tumor suppressor gene in different types of tumors, and it is also a useful clinical prognostic biomarker (Wang et al., 2014). In IDD tissues, the level of miR-31-5p, miR-124 A, and miR-127-5p is generally down-regulated (Wang et al., 2019). It is interrelated to the role of ECM synthesis in hypertrophic scar formation (Wang et al., 2017). This study confirmed the abnormal regulation of miR-31-5p in NP tissues of IDD patients. Subsequently, we obtained degenerated human NP cells from IDD patients to study the pathways through which miR-31-5p functions in IDD.

## Materials and Methods

### Patient Samples

Nucleus pulposus samples were taken from 82 underwent discectomy patients with degenerative disc disease (57.6 ± 5.3 years in total). The indications for surgery are failure of conservative treatment and progressive neurological deficits, such as progressive motor weakness or cauda equina syndrome. Here we exclude patients with lumbar spine stenosis, ankylosing spondylitis, isthmus or degenerative spondylolisthesis, or diffuse idiopathic skeletal hypertrophy. A total of 91 patients were recruited to be used as the age- and sex-matched controls, which involved collecting NP tissue samples taken from fresh traumatic lumbar fracture as the result of anterior decompressive surgery. Before surgery, these patients underwent a routine lumbar spine MRI scan. According to Pfirrmann classification, the degree of IDD was graded. The research protocol has been approved by the Ethics Committee of The Third Xiangya Hospital Affiliated to Central South University, and the written informed consent of each participant has been obtained.

### IDD Model

In this study, an IDD model was established in mice (12 weeks old) by AF needle puncture (Ji et al., 2015; Xia et al., 2015). We selected the tail disc for establishment of an IDD model because of its anatomical accessibility and minimal surgical morbidity (O’Connell et al., 2007; Beckstein et al., 2008; Schaer et al., 2012). Ketamine (100 mg/kg) was selected as the anesthetic for mice in the operation group, and they were injected intraperitoneally. After the general anesthesia effect was achieved, the mouse was fixed in the left side position and executed the model operation. A sagittal small skin incision was performed from Co6 to Co8 to help locate the disc position for needle insertion in the tail. Subsequently, Co6–Co7 coccygeal discs were punctured using a syringe needle. The syringe needle was inserted into Co6–Co7 disc along vertical direction and then rotated in the axial direction by 180° and held for 10 s. Using a 31-G needle, puncture the NP through the AF parallel to the end plate through the NP, and then insert it into the 1.5 mm disc to decompress the nucleus. The other parts remain unchanged as the comparison part. For treatment experiments, intervertebral discs were harvested from WT mice at 6 and 12 weeks after surgery and then studied.

### Primary Nucleus Pulposus Monolayer Culture

The NP tissues were washed three times with phosphate-buffered saline (PBS; Gibco, Grand Island, NY, United States), minced into small fragments and digested in 0.25% (w/v) trypsin (Gibco) and 0.2% (w/v) type Π collagenase (Gibco) and then placed in PBS for approximately 3 h at 37°C in a gyratory shaker. Cells were filtered through a 70 μm mesh filter (BD, Franklin Lakes, NJ, United States). Primary NP cells were cultured with growth medium (Dulbecco’s Modified Eagle’s Medium) and Ham’s F-12 Nutrient Mixture (DMEM-F12; Gibco), 20% (v/v) fetal bovine serum (FBS; Gibco), 50 U/mL penicillin, and 50 μg/mL streptomycin (Gibco) in 100 mm culture dishes in a 5% (v/v) CO_2_ incubator. The cells were passaged at approximately 80% (v/v) confluence using trypsin and subcultured in a 60 mm culture dish (2.5 × 105 cells/well). Cells that had been passaged no more than twice were used in the subsequent experiments.

### MiR-31-5p NP Generation and Injection

Use Silencer^®^ small interfering RNA (siRNA) labeling kit (#AM1636) to transfect cultured primary human NP cells with Cy3 labeled or unlabeled miR-31-5p mimics or inhibitors. Use Lipofectamine RNAiMAX Transfection Reagent (Invitrogen) at 50 nM. To inhibit the level of SDF-1/CXCR7, cells were transfected with siRNA (Thermo Scientific Dharmacon^®^) using Lipofectamine 3000 (Invitrogen) according to the manufacturer’s instructions. For treatment experiments, we divided 48 male mice (12-week-old C57BL/6) (Bar Harbor Jackson Laboratory, United States) that underwent IDD surgery into four groups (12 mice in each group). In the Cy3 simulation control group NP treatment group or Cy3-miR-31-5p simulation NP treatment group, mice were locally injected with 20 μL Cy3-miR simulation control NP or 20 μL Cy3-miR-31-5p simulation NP on the 1, 7, and 14 days. In order to determine the transfection efficiency of the Cy3-labeled miR-31-5p mimic/inhibitor or its negative control, IVIS 200 imaging system (Xenogen, Calper Life Science, MA, United States) was applied for *in vivo* fluorescence imaging, and at different times Perform histological examination at points after each group injection (24, 48, and 72 h). In the 6th and 12th weeks after the operation, the intervertebral discs were collected for histological and radiological evaluation of each group.

### Histological and Radiographic Evaluation

The intervertebral discs of the mice were fixed in 10% neutral formalin buffer for 1 week, and then soaked in EDTA decalcification solution for decalcification. Then it was embedded and sectioned. Then the histological images were analyzed by hematoxylin, eosin and saffron O-type green staining using Olympus BX51 microscope (Olympus Center Valley, PA, United States). Based on a literature review of research on IDD, an improved histological grading system was developed (Horner et al., 2002; Boos et al., 2003; Masuda et al., 2005; Rousseau et al., 2007; Han et al., 2008; Lipson and Muir, 2010; Tam et al., 2018). More specifically, the cellularity and morphology of the AF, NP, and the border between the two structures were examined. The scale is based on five categories of degenerative changes with scores ranging from 0 points (0 in each category) for a normal disc to 15 points (three in each category) for a severely degenerated disc. For morphology of the NP, score 0: round shape and the NP constitutes >75% of the disc area, score 1: round shape and the NP constitutes 50–75% of the disc area, score 2: round shape and the NP constitutes 25–50% of the disc area, score 3: round shape and the NP constitutes <25% of the disc area. For cellularity of the NP, score 0: stellar-shaped cells with a proteoglycan matrix located at the periphery, evenly distributed, score 1: partially stellar and partially round cells, more stellar than round, score 2: mostly large, round cells, separated by dense areas of proteoglycan matrix, score 3: large, round cells, separated by dense areas of proteoglycan matrix. For morphology of the AF, score 0: well-organized collagen lamellae with no ruptures, score 1: inward bulging, ruptured, or serpentine fibers constitute <25% of the AF, score 2: inward bulging, ruptured, or serpentine fibers constitute 25-50% of the AF, score 3: inward bulging, ruptured, or serpentine fibers constitute >50% of the AF. For cellularity of the AF, score 0: fibroblasts comprise >90% of the cells, score 1: fibroblasts comprise >75–90% of the cells, score 2: intermediate, score 3: chondrocytes comprise >75% of the cells. For border between the NP and AF, score 0: normal, without any interruption, score 1: minimal interruption, score 2: moderate interruption, score 3: severe interruption.

Radiographs were taken at 6 and 12 weeks after the injection. After the last radiography, MRI was performed on all mice using a 7.0 T animal-specific MRI system (Bruker Pharmascan, Ettlingen, Germany). T2-weighted sections in the median sagittal plane were obtained using the following settings: a fast spin echo (SE) sequence with a time to repetition (TR) of 3,000 ms and a time to echo (TE) of 70 ms; the slice thickness was 0.5 mm, and the gap was 0 mm. Pfirrmann classification was used to assess the degree of IDD degeneration. The average score of the punctured IDD was calculated as the amount of degeneration for each mouse. The change in IVD height was evaluated by the disc height index (DHI). Measurements of internal control discs were carried out together with their corresponding punctured discs. Disc height and the adjacent vertebral body heights were measured from the midline as 25% of the disc’s width from the midline on either side. The DHI was expressed as the mean of the three measurements from midline to the boundary of the central 50% of disc width divided by the mean of the two adjacent vertebral body heights. Changes in the DHI of punctured discs were expressed as a percentage (% DHI = post-punctured DHI/pre-punctured DHI × 100).

### qRT-PCR

Total RNA was extracted from the transfected cell lines using Trizol reagent (Takara, Japan). One-step PrimeScript miRNA cDNA Synthesis Kit (Takara) was applied for reverse transcription. We applied SYBR Geen Realtime PCR Master Mix (Takara) to perform qRT-PCR and synthesize data on the ABI 7300 system (ABI). In addition, U6 snRNA were applied as internal controls.

### CpG Island Prediction and Bisulfite Sequencing PCR

The promoter region was predicted by using Promoter Inspector prediction software^1^. The CpG prediction algorithm was applied to predict the CpG islands associated with the promoter. Genomic DNA was isolated from NP by Qiagen DNeasy Blood and Tissue Kit, and then placed in bisulfite. Then it was amplified with bisulfite sequencing PCR (BSP) primers and cloned into pGEMT Easy vector (Promega, WI, United States). The samples are then sequenced and the data is analyzed by BIQ analyzer.

### Microarray Analyses

First, the Trizol method was employed to extract total RNA from NP cells preserved with a final concentration of 1 mg ml^–1^. Then the mi RNA isolation kit (Ambion) of mirVana^TM^ was employed to purify the mi RNA part of the total RNA, and finally the extracted mi RNA samples were analyzed on the chip, using the human mi RNA chip (v.12.0) of Agilent Company for analysis. Hybridization. Data processing was performed by GeneSpring GX v12.1 software package (Agilent Technologies).

### Transfection

We applied miR-31-5pmimic, miR-31-5p inhibitor, non-targeting siRNA control (si-NC) and miRNA control (RiboBio Inc., Guangzhou, China) according to the instructions of the Lipofectamine^TM^ 3000 kit (Invitrogen, Carlsbad, CA, United States) transfected into Cy3 labeled or unlabeled NP cells.

### Luciferase Assay

The cells were planted in a 24-well plate at 105 cells/well and cultivated for 24 h. According to the instructions of the detection kit (Promega), the cells were transfected with the luciferase reporter gene level plasmid, and the miR-31-5p mimic and the control group were given at the same time. After 24 h, the luciferase activity was measured.

### Flow Cytometer

Cell apoptosis was evaluated using FITC-Annexin V and ethidium iodide (PI, 556547, BD biosciences). For the operation method, refer to the kit instructions and calculated the percentage of apoptosis according to the fluorescence intensity.

### EdU Analysis

After processing the cells according to the treatment conditions and time of each group, 50 μmol/L Edu (Sigma-Aldrich) medium was replenished to each dish. The cells were cultured in a 37°C, 5% CO_2_ incubator for 2 h, and rinsed with PBS twice. The cells were fixed with 4% paraformaldehyde, 0.2% glycine was replenished, and rinsed with PBS for 5 min. The membrane was ruptured by 0.5% Triton-100 for 10 min, rinsed with PBS, and 100 μL Apollo staining reaction solution was replenished to each well. It was incubated on a shaker at room temperature in the dark for 30 min, rinsed with 0.5% Triton-100, and then rinsed with 100 μL methanol and PBS. It was stained with DAPI for 20 min, then rinsed with PBS, and the results were observed under a fluorescence microscope.

### Fluorescence *in situ* Hybridization

Locked nucleic acid (LNA) probes complementary to miR-31-5p are labeled with 5′ and 3′-digoxigenin (Exiqon, Woburn, MA, United States). The NP tissue of IDD patients is employed for FISH detection. This section was taken out, and then the gene break probe was dropped. Then, it was placed on the hybridization instrument at 75°C for 10 min, 42°C overnight. It was taken out the next day, and rinsed at room temperature for 5 min and 72°C for 3 min. It was dried and DAPI was replenished dropwise to cover the slide. The fluorescence microscope (Olympus IX-81; Olympus, Tokyo, Japan) was employed to read and take the image. The intensity of miR-31-5p staining was scored from 0 to 4 based on no staining (Jiang et al., 2013). Then analyzed the miR-31-5p cells in three representative high-power fields of a single sample.

### Bioinformatics Analysis

Gene Ontology (GO) analyses, with terms as biological processes, molecular function, and cellular component, were performed to predict the influences of the downregulated mRNAs on intervertebral discs using the DAVID bioinformatics program^2^. Cytoscape software (v.3.6.1) was used to create and analyze a miRNA-hub gene network to explore the relation between potential target genes and candidate DE-miRNAs. The potential target genes of miR-31-5p were predicted by TargetScan^3^, miRanda^4^, PicTar^5^, PITA^6^, and RNA22^7^.

### Western Blot

Collect cells in the logarithmic growth phase and seed them in a petri dish with a diameter of 60 mm. After 24 h of cell treatment, cells of each group were collected and employed RIPA lysate. The protein concentration of each group of cells was evaluated by BCA method, and 6–12% SDS-PAGE electrophoresis was executed with 20 μg/well of protein. The proteins separated by electrophoresis are transferred to the PVDF membrane. After incubating the first antibody, the second antibody anti-rabbit immunoglobulin G (ab99697) was incubated at room temperature for 1 h, and developed with ECL reagent (Thermo Fisher Scientific, Inc.). Use ImageJ software (National Institutes of Health) to analyze the gray value of each protein band to determine the optical density.

The primary antibody information is as follows: anti-Col II antibody (Abcam: ab34712), anti-agroglycan antibody (ab36861), anti-ADAMTS-5 antibody (ab41037), anti-MMP13 antibody (ab39012), anti-SDF-1 antibody (ab155090), anti-CXCR7 antibody (ab72100), anti-β-actin (ab150301).

### Cellular Immunofluorescence

The cells were seeded on glass slides in a 6-well culture plate, transfected the next day, and 4% paraformaldehyde was employed to fix the cells on the glass slides 48 h later. Then it was permeabilized with 0.5% Triton X-100 for 20 min, blocked with normal goat serum for 30 min, and the primary antibody was dropped and incubated overnight at 4°C. These cells were incubated with FITC-labeled secondary antibody in the dark for 1 h at room temperature. The nuclei were counterstained with DAPI, rinsed, mounted and observed under a fluorescence microscope.

The primary antibody information is as follows: anti-Col II antibody (Abcam: ab34712), anti-agroglycan antibody (ab36861), anti-ADAMTS-5 antibody (ab41037), anti-MMP13 antibody (ab39012). The secondary antibody is goat anti-rabbit IgG (Abcam: ab150077).

### Immunofluorescence and TUNEL Staining of Tissue Sections

The frozen part of the mouse dish was fixed with 4% paraformaldehyde. The tissue sections were taken out, and after deparaffinization and hydration, they were fixed in pre-cooled 40 g/L paraformaldehyde for 5 min. It was treated with proteinase K for 10 min, immersed in a buffer solution containing 5% hydrogen peroxide for 20 min to block the endogenous peroxidase activity. TUNEL (Invitrogen) reaction mixture was replenished and reacted at 37°C for 1 h. POD conversion solution was replenished and reacted at 37°C for 30 min. Then, DAB was developed and the apoptosis was observed under the microscope.

### Statistical Analysis

All data were analyzed using SPSS 19.0 software (SPSS Inc., Chicago, IL, United States), and the experiment was repeated three times. The result is expressed as *x* ± SD. The comparison between the two groups was executed using an independent sample *t*-test. Multiple group comparisons were performed through one-way analysis of variance. *P* < 0.05 was accounted significant.

## Results

### miR-31-5p Declined in NP Tissues of IDD Patients

**FIGURE 1:**
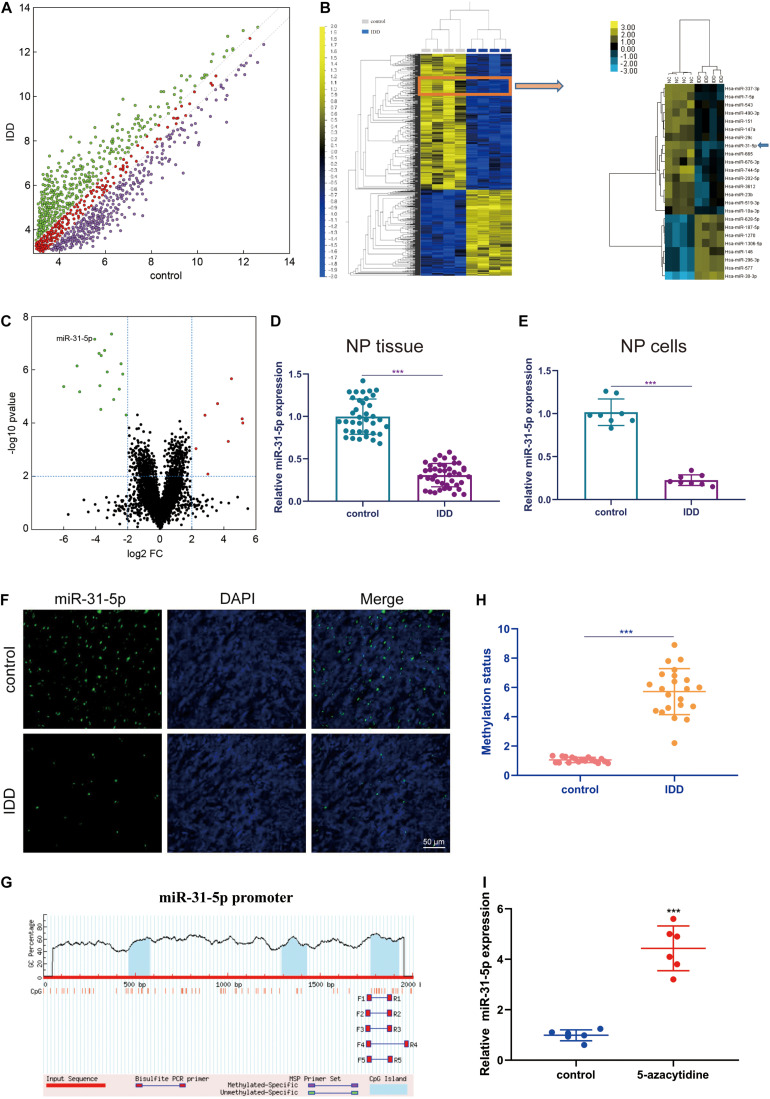
miR-31-5p declined in NP tissues of IDD patients. **(A)** Represents the scatter plot of miRNA expression profiles between IDD patients and the control group (green dots indicate more than twofold increase; purple dots indicate more than twofold down). **(B)** Describes the heat maps of 24 differentially expressed miRNAs. **(C)** The volcano graph represents the difference in miRNA expression levels between IDD patients and controls. The *y*-axis is the negative *P*-value after adjusting Log10, and the *x*-axis represents the multiple change of Log2. The red dots on the right represent up-regulated miRNAs, and the green dots on the left represent down-regulated miRNAs. miR-31-5p is indicated. **(D)** The miR-31-5p level in human nucleus pulposus tissue was evaluated through qRT-PCR. **(E)** The miR-31-5p level in human nucleus pulposus cells was valued through qRT-PCR. **(F)** FISH analysis was executed on IDD patients and the control group. Scale bar = 50 μm. **(G)** Methylation of miR-31-5p promoter region. **(H)** Methylation status of IDD patients and controls. **(I)** The PCR result revealed that 5-azacytidine (DNA methyltransferases inhibitor) can effectively restore miR-31-5p expression. *n* = 3. IDD, intervertebral disc degeneration; miR, microRNA; FC, fold change; DAPI, 4′,6-diamidino-2-phenylindole. ^∗∗∗^*P* < 0.001.

The occurrence of IDD is closely interrelated to the dysregulation of interrelated miRNAs (Wang et al., 2015). In order to study the part of miRNAs in IDD, we evaluated miRNAs by microarray (Figure 1A). Then, these significantly dysregulated miRNAs were subjected to unsupervised cluster analysis to differentiate IDD patients from controls (Figures 1B,C). From Figures 1B,C, it can be observed that miR-31-5p has a significant imbalance. Therefore, we chose miR-31-5p for further research. We used further qRT-PCR experiments to assess miR-31-5p level in human nucleus pulposus tissues and nucleus pulposus cells. The results demonstrated that compared to the control group, the miR-31-5p in the nucleus pulposus tissue and nucleus pulposus cells of the IDD group was significantly reduced (Figures 1D,E, *P* < 0.001). We confirmed this conclusion through further fluorescence *in situ* hybridization experiments (Figure 1F). In order to probe the upstream mechanism of miR-31-5p down-regulation in NP, CpG islands in the miR-31-5p promoter region were predicted (Figure 1G). As we can see, the methylation status of IDD group was significantly higher than that of control group (Figure 1H, *P* < 0.001). The PCR result revealed that 5-azacytidine (DNA methyltransferases inhibitor) can effectively restore miR-31-5p expression (Figure 1I). From the above results, it was revealed that the level of miR-31-5p in the NP tissue of IDD patients declined and the methylation status increased.

### The Effect of miR-31-5p Overexpression or Silence on the Phenotype of NP Cells

**FIGURE 2:**
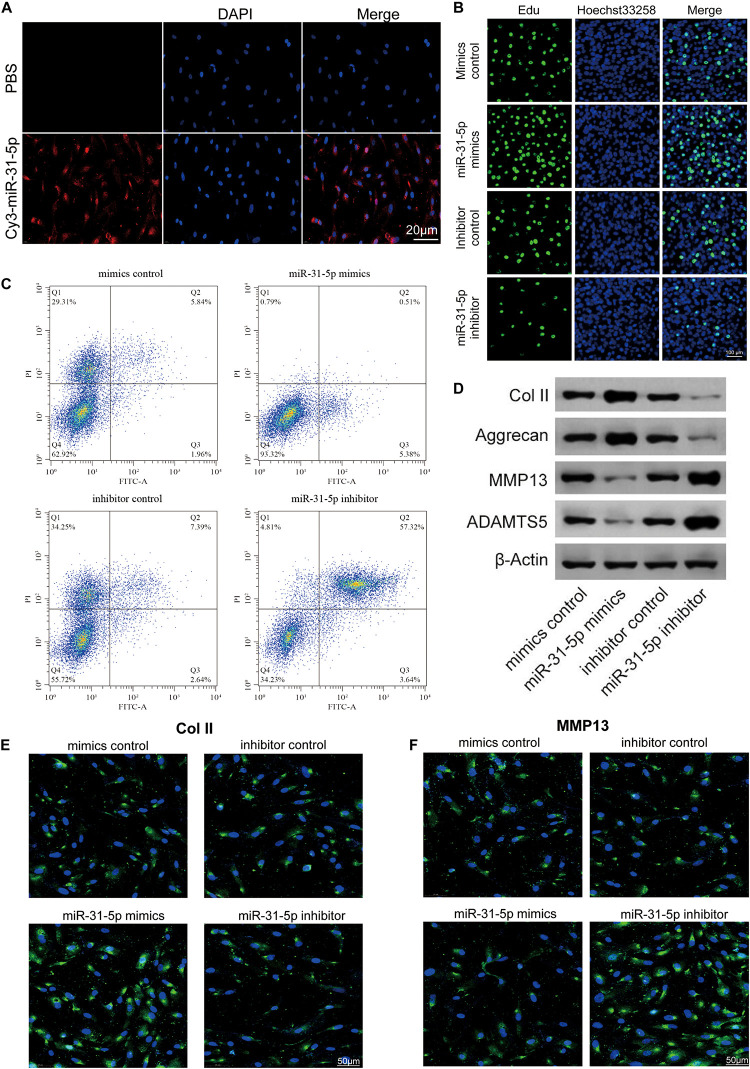
The effect of miR-31-5p overexpression or silence on the phenotype of NP cells. **(A)** Cy3 was employed to detect miR-31-5p transfected and cultured NP cells, scale bar = 20 μm. **(B)** Analyze the proliferation of NP cells transfected with different treatments by EdU, scale bar = 100 μm. **(C)** The apoptosis of NP cells was analyzed by Flow cytometer. **(D)** The level of MMP13, Col II, Aggrecan, and ADAMT5 was valued through western blot. **(E,F)** The Col II and MMP13 levels were evaluated through immunofluorescence. *n* = 3. IDD, intervertebral disc degeneration; miR, microRNA; PBS, phosphate buffer saline; DAPI, 4′,6-diamidino-2-phenylindole; FITC, fluorescein isothiocyanate; Col II, type II collagen; MMP, matrix metalloprotein; ADAMTS, a disintegrin-like and metalloproteinase with thrombospondin motifs; NP, nucleus pulposus; EdU, 5-Ethynyl-2′-deoxyuridine.

Previous studies have demonstrated that miR-31-5p is closely interrelated to the occurrence of IDD (Yang et al., 2019). To further probe the part of miR-31-5p in IDD, the miR-31-5p mimic or inhibitor was transfected into primary human NP cells. It was demonstrated that the transfection efficiency of Cy3-labeled miRNA was evaluated (Figure 2A). We further studied the effects of miR-31-5p overexpression or silencing on NP cell proliferation, apoptosis, ECM formation, and matrix-degrading enzymes. The results of EdU demonstrated that compared with miR-31-5p inhibitor, the upregulation of miR-31-5p level promoted NP cell proliferation (Figure 2B). In terms of apoptosis, upregulation of miR-31-5p level restrained NP cell apoptosis (Figure 2C). We further evaluated the function of miR-31-5p levels on anabolic/catabolism markers through the function gain and loss of function studies. It was demonstrated that the levels of Col II and Aggrecan increased in primary human NP cells transfected with miR-31-5p mimics. In primary human NP cells transfected with miR-31-5p inhibitor, the levels of ADAMTS-5 and MMP13 increased (Figure 2D). We further verified this function by immunofluorescence (Figure 2E,F). Overall, the data signifies that the overexpression of miR-31-5p facilitates the synthesis and proliferation of NP cell matrix.

### The Relevance of miR-31-5p to SDF-1/CXCR7 Axis

**FIGURE 3:**
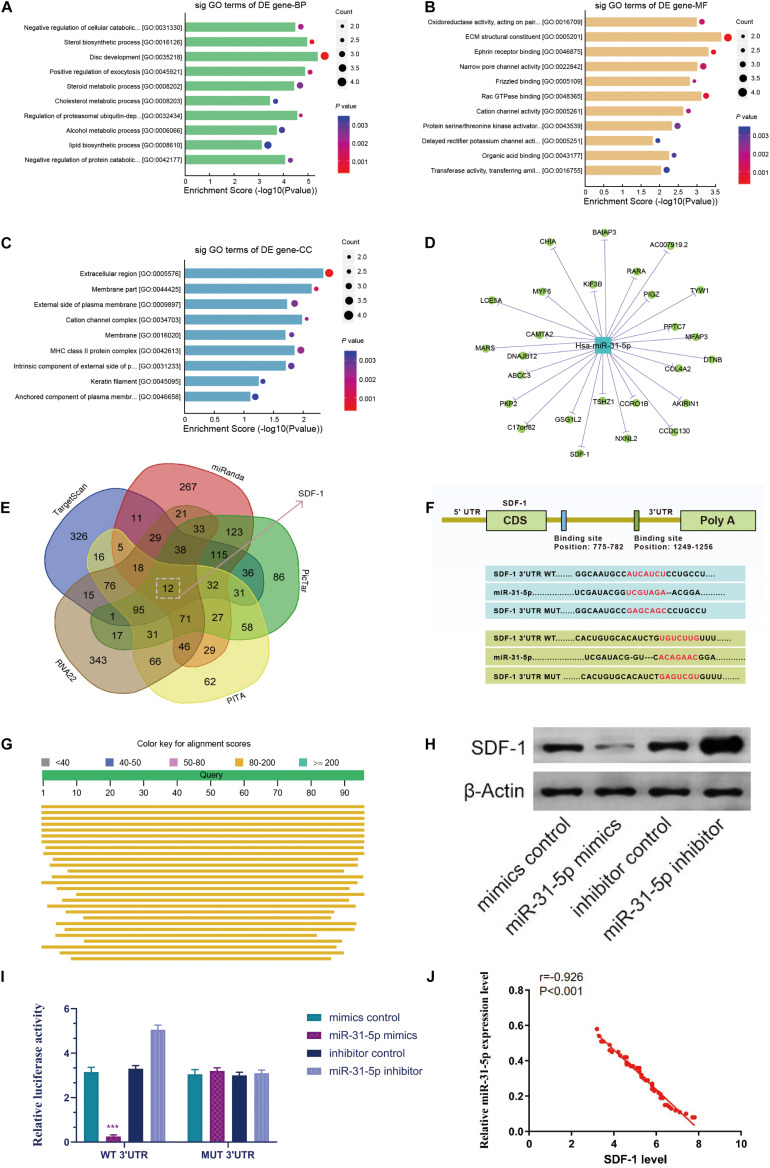
The relevance of miR-31-5p to SDF-1/CXCR7 axis. **(A–C)** For biological processes, molecular functions and cellular components, the down-regulated GO term with the highest p value. **(D)** The target of miR-31-5p was verified by Cytoscape. **(C)** The Venn diagram predicts that SDF-1/CXCR7 axis is the target of miR-31-5p. **(E)** Venn diagram displaying miR-31-5p computationally predicted to target SDF-1 by different algorithms. **(F)** The mRNA 3′UTR of SDF-1 and the putative miR-31-5p binding site sequence has high sequence conservation and complementarity with miR-31-5p. **(G)** miR-31-5p is highly conservative. **(H)** The wild-type or mutant SDF-1 3′UTR reporter plasmid and miR-31-5p mimic or inhibitor are co-transfected into human NP cells. **(I)** The expression level of SDF-1 was valued through western blot. *n* = 3. **(J)** The miR-31-5p expression level was negatively correlated with SDF-1 in IDD (*r* = –0.926, *P* < 0.001). IDD, intervertebral disc degeneration; miR, microRNA; NP, nucleus pulposus; GO, Gene Ontology; mut, mutant; WT, wild-type; SDF-1, stromal cell-derived faceor-1; CXCR, C-X-C chemokine receptor. ^∗∗∗^*P* < 0.001.

We performed a GO analysis on the dysregulated mRNA. Our results demonstrated that in the biological process, the GO term of the down-regulated genes with the highest *P*-value among the molecular functions and cellular components is interrelated to disc development (GO: 0035218), ECM structural components (GO: 0005201), and extracellular regions (GO: 0005576) (Figures 3A–C). In addition, we have constructed a miRNA-mRNA network map through Cytoscape software (Figure 3D). To further probe the potential targets of miR-31-5p, we compiled all the predicted genes into a Venn analysis map (Figure 3E). According to the result, we demonstrated that the SDF-1/CXCR7 axis is the target of miR-31-5p (Figure 3F). Besides, miR-31-5p is proven to be highly conserved among species (Figure 3G). To further verify the association between the SDF-1/CXCR7 axis and miR-31-5p, luciferase reporter gene analysis was employed to test the association between them. The results demonstrated that the relative luciferase reporter activity of wild-type (WT) co-transfected with miR-31-5p mimic in primary human NP cells was meaningfully lower than that of mutant (mut) cells transfected with miR-31-5p mimic (Figure 3H, *P* < 0.001). We further verified this result at the protein level. The results of western blot demonstrated that the level of SDF-1 protein in miR-31-5p mimic group declined (Figure 3I). The miR-31-5p expression level was negatively correlated with SDF-1 in IDD (*r* = −0.926, *P* < 0.001, Figure 3J). The above results indicate that the SDF-1/CXCR7 axis is the target of miR-31-5p.

### miR-31-5p Level Regulated IDD via SDF-1/CXCR7 Axis

**FIGURE 4:**
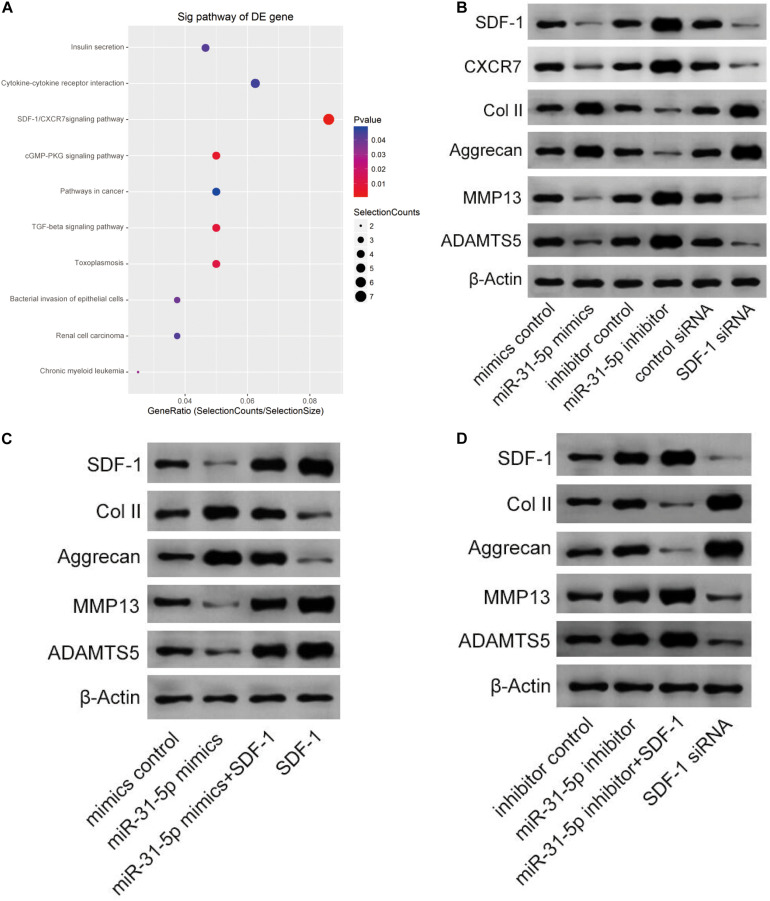
miR-31-5p expression regulates IDD via SDF-1/CXCR7 axis. **(A)** The IDD-rich SDF-1/CXCR7 pathway was analyzed by KEGG. **(B)** The expression level of SDF-1, CXCR7, Col II, Aggrecan, MMP13, and ADAMT5 was valued through western blot. **(C)** The expression level of SDF-1, Col II, Aggrecan, MMP13, and ADAMT5 was valued through western blot. **(D)** The expression level of SDF-1, Col II, Aggrecan, MMP13, and ADAMT5 was valued through western blot. IDD, intervertebral disc degeneration; miR, microRNA; Col II, type II collagen; MMP, matrix metalloprotein; ADAMTS, a disintegrin-like and metalloproteinase with thrombospondin motifs; KEGG, Kyoto Encyclopedia of Genes and Genomes; SDF-1, stromal cell-derived faceor-1; CXCR, C-X-C chemokine receptor.

As shown in Figure 4A, the SDF-1/CXCR7 signaling pathway is significantly rich in genes and genomic pathways of the Kyoto Protocol. Through further research, we confirmed that miR-31-5p regulates IDD through the SDF-1/CXCR7 axis pathway. We transfected the cultured primary human NP cells with miR-31-5p mimic, miR-31-5p inhibitor, or its negative control. Western blot results demonstrated that in NP cells, the protein levels of SDF-1, CXCR7, ADAMTS-5, and MMP13 of miR-31-5p mimics declined, while in NP cells transfected with miR-31-5p inhibitor, SDF-1, CXCR7, ADAMTS-5, and MMP13 protein levels increased. In addition, the effects of SDF-1 siRNA on SDF-1, CXCR7, ADAMTS-5, and MMP13 are similar to those induced by miR-31-5p mimics. This manifests that miR-31-5p regulates IDD through the SDF-1/CXCR7 axis pathway (Figure 4B). Further experiments were executed to verify the association between miR-31-5p and SDF-1/CXCR7 axis (Figures 4C,D). The above experimental results show that miR-31-5p acts through the SDF-1/CXCR7 axis pathway.

### Upregulation of miR-31-5p Level Prevented IDD Development

**FIGURE 5:**
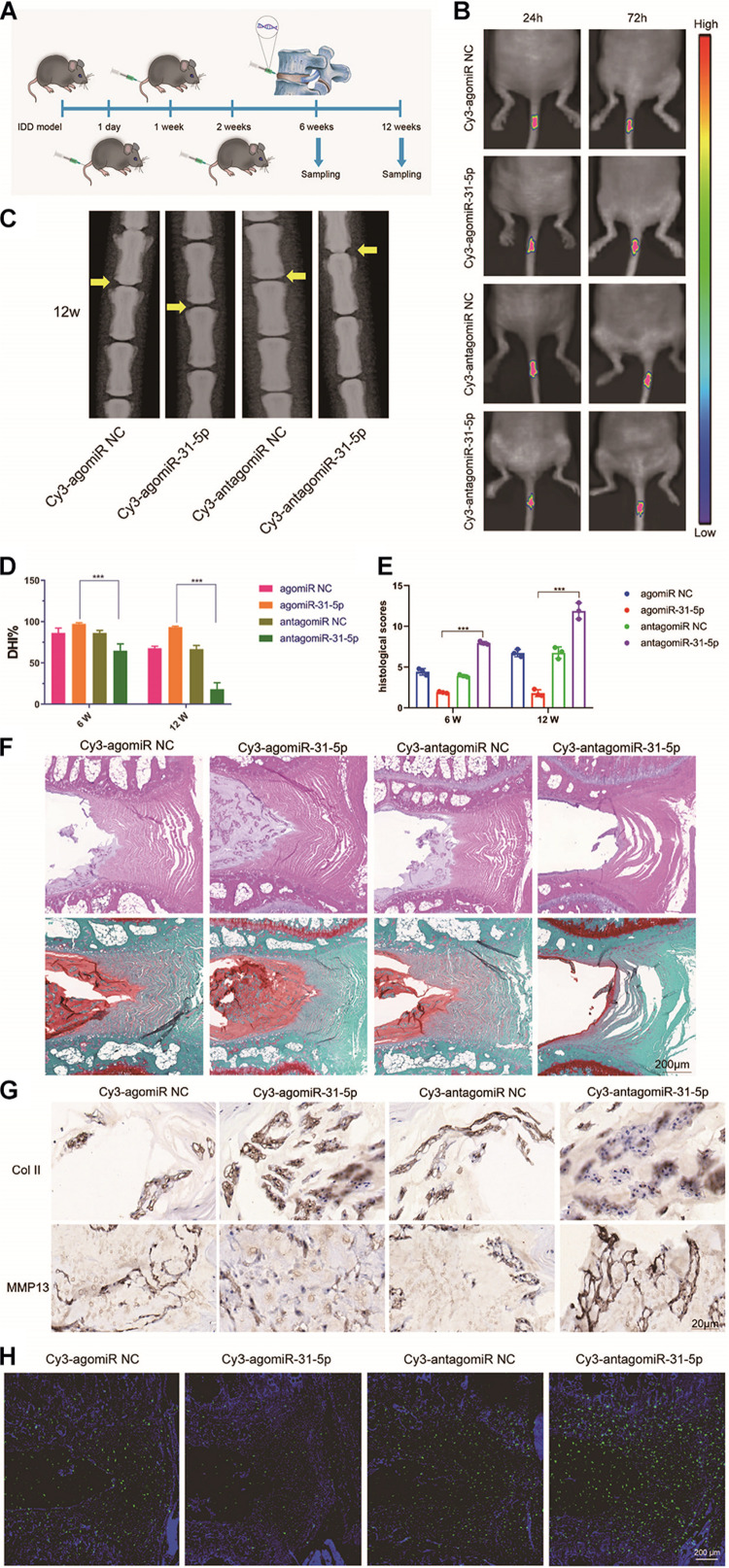
Upregulation of miR-31-5p expression prevented IDD development. **(A)** Experiments in which miR-31-5p mimics, miR-31-5p inhibitors, or their negative controls were injected at 1, 1, and 2 weeks after surgery. **(B)** Time-dependent fluorescence images of mice treated with Cy3-miR-31-5p for 24 and 72 h. From blue to red represents the change in fluorescence signal intensity from low to high. **(C)** X-ray evaluation of intervertebral disc degeneration. **(D–F)** The DHI of 6 and 12 W under different treatments and the changes in histology were evaluated. The histological findings after IDD surgery were a loss of NP cells and their replacement by cells of a more fibroblast-like phenotype. Increased numbers of NP cells were observed in the group treated by agomiR-31-5p compared to the group with antagomiR-31-5p. **(G)** Immunostaining of Col II and MMP13 in IDD model treated with miR-31-5p NP. Scale bar = 20 μm. **(H)** TUNEL staining was executed on the intervertebral disc to determine the apoptotic activity, scale bar = 200 μm. IDD, intervertebral disc degeneration; NC, negative control; miR, microRNA; Col II, type II collagen; MMP, matrix metalloprotein. ^∗∗∗^*P* < 0.001.

We further studied the part of miR-31-5p in IDD and the molecular mechanisms involved. We induced the IDD model by WT mice, and then injected miR-31-5p mimic or inhibitor NP and control NP locally on 1, 7, and 14 days after surgery (Figure 5A). We monitored the *in vivo* targeting ability of NP in real time. The results revealed that miR-31-5p mediated by NPs revealed a good delivery function in mice (Figure 5B). In order to further probe the part of miR-31-5p in IDD, we conducted further tests through radiography and histological evaluation. The results revealed that compared with the control group, the local delivery of miR-31-5p mimic NPs significantly protected the IVD structure, which indicated that miR-31-5p overexpression had a protective function on the surgically induced IDD model (Figures 5C–F). NPs treated with miR-31-5p mimic significantly reduced the level of MMP13, while the level of col II increased. The miR-31-5p inhibitor group had the opposite effect (Figure 5G). We tested the apoptosis of NP cells after different treatments, and the results of TUNEL staining revealed that NP cell apoptosis was significant in mice treated with miR-31-5p mimic NPs. Reduce (Figure 5H). The above results show that overexpression of miR-31-5p has a significant effect on the treatment of IDD, which indicates that miR-31-5p is a potential therapeutic target for IDD.

## Discussion

Many studies have manifested that NP cells are essential for maintaining the structural integrity of intervertebral discs, and the phenotype of NP cells is closely interrelated to the pathogenesis of IDD (Jiang et al., 2013; Chen et al., 2018). Regarding the current treatment methods and methods, in-depth research is needed to promote the improvement of prevention and treatment methods. Recent studies have reported that certain specific miRNAs have been identified as important regulatory factors and biomarkers of IDD, which are important substances for the prevention and treatment of IDD (Xu et al., 2016; Cai et al., 2017). The underlying mechanism is to regulate certain chemokines and transcription and translation in cells through specific miRNAs, and then regulate the cell phenotype (Wang et al., 2015; Ji et al., 2018; Sherafatian et al., 2019).

Microarray detection of IDD NP was executed to analyze the differential miRNA level profile between IDD and normal tissues. miR-31-5p is closely interrelated to the occurrence and development of many diseases (Chen et al., 2019; Gupta et al., 2019; He et al., 2020). In this study, the part of miR-31-5p in the occurrence and development of IDD was further verified. In order to further probe the imbalance of miR-31-5p in IDD, the level of miR-31-5p was evaluated by qRT-PCR, and it was demonstrated that miR-31-5p was down-regulated in IDD. Further FISH maps and methylation conditions also verified this result. In order to clarify the function of miR-31-5p level on NP cells, the function of miR-31-5p level and silencing miR-31-5p on the phenotype of NP cells was studied by regulating the level of miR-31-5p. The results proved that overexpression of miR-31-5p is interrelated to increased proliferation of NP cells, inhibition of apoptosis, increased ECM formation, and inhibition of matrix degrading enzymes. These phenotypic changes are the key biological process of IDD. The increase in ECM synthesis is manifested by the increase in the ingredients of Col II and Aggrecan, while the decrease of the ingredients in ADAMTS-5 and MMP13 (Gendron et al., 2007; Takaishi et al., 2008). Therefore, miR-31-5p is involved in the pathogenesis of IDD.

Stromal cell-derived factor 1 is expressed in various tissues, such as lung, liver, bone marrow, and lymph nodes. CXCR7 is receptor for SDF-1 and binds to SDF1 specifically to form the SDF1/CXCR7 axis. SDF-1/CXCR7 is closely interrelated to the occurrence of many diseases, and previous studies have demonstrated that SDF-1/CXCR7 is interrelated to the occurrence of IDD (Luo et al., 2018; Hain, 2019; Zhang et al., 2019). Once SDF-1 binds to CXCR7, it induces NP cells to release various enzymes (MMP13 and ADAMTS5), leading to the degradation of type II collagen and proteoglycan. In our study, it is further verified that miR-31-5p regulates the changes of NP phenotype through the SDF-1/CXCR7 axis pathway. It is consistent with that in NP cells, the overexpression of miR-31-5p is consistent with the reduction of protein levels of SDF-1, CXCR7, ADAMTS-5, and MMP13. Silencing miR-31-5p is consistent with increased protein levels of SDF-1, CXCR7, ADAMTS-5, and MMP13. The effects of SDF-1 siRNA on SDF-1, CXCR7, ADAMTS-5, and MMP13 are similar to those induced by miR-31-5p mimics. This result also indicates that there is a negative regulatory association between miR-31-5p and SDF-1/CXCR7 axis. SDF-1 can offset the function of overexpression of miR-31-5p on the protein levels of SDF-1, col II, Aggrecan, ADAMTS-5, and MMP13. It is worth noting that the function of SDF-1 on miR-31-5p inhibition is limited to col II and Aggrecan. The above information manifests that miR-31-5p regulates IDD through the SDF-1/CXCR7 axis pathway. Although other study had reported some critical biological functions of miR-31-5p, this was the first time to reveal its function on SDF1/CXCR7 axis.

The important clinical significance of miR-31-5p and its downstream pathways was further confirmed by *in vivo* examination using an inducible IDD animal model. As expected, in terms of protecting the phenotype of NP cells, overexpression of miR-31-5p effectively alleviated the symptoms of IDD. Therefore, as an effective regulator of IDD, miR-31-5p has therapeutic potential. It is not the first report that miRNAs play a positive role in the treatment of IDD. Many previous studies have manifested that miRNAs regulate IDD through β-catenin, MMP, apoptosis and cell proliferation (Li et al., 2012; Liu et al., 2013; Wang et al., 2011; Wei et al., 2013; Yu et al., 2013; Feng et al., 2018; Ge and Li, 2019). It is worth emphasizing that the level of miRNAs may be regulated by the methylation of the promoter region, which may affect certain phenotypes (Augoff et al., 2012; Li et al., 2020). In this research, according to the prediction of CpG islands in the promoter, three strong CpG islands were identified in miR-31-5p interrelated promoters. This makes us hypothesize that miR-31-5p in its host gene may be regulated by promoter methylation. Further experiments proved that hypermethylation may contribute to the loss of miR-31-5p in NP cells. In addition, a comprehensive understanding of the relevant molecular mechanisms will have a function on the efficacy of treatment.

In addition to the important findings of this study, restrictions on the scope of the study still exist. First, although the function of down-regulation of miR-31-5p on the phenotype of NP cells has been demonstrated, the mechanism of down-regulation of miR-31-5p is not fully understood. Secondly, other potential effects of manually increasing miR-31-5p are not yet known. These issues may be further studied in follow-up research work.

In conclusion, miR-31-5p reduces IDD by targeting SDF-1/CXCR7 to regulate cell proliferation, apoptosis, and ECM degradation. These findings lay the foundation for follow-up research and the understanding and understanding of IDD, and at the same time provide a promising therapeutic target for IDD treatment.

## Conclusion

In this study, our results indicate that miR-31-5p has a potential part in the proliferation, apoptosis, ECM formation of NPs, and matrix degrading enzymes in NP cells. These effects may be achieved through the SDF-1/CXCR7 axis, and further *in vitro* experiments have further verified the part of miR-31-5p. Importantly, our results provide evidence for miR-31-5p as a potential target, diagnostic indicator and prognostic indicator for IDD patients.

## Data Availability Statement

The raw data supporting the conclusions of this article will be made available by the authors, without undue reservation.

## Ethics Statement

The studies involving human participants were reviewed and approved by The Third Xiangya Hospital of Central South University. The patients/participants provided their written informed consent to participate in this study. The animal study was reviewed and approved by The Third Xiangya Hospital of Central South University.

## Author Contributions

All authors listed have made a substantial, direct and intellectual contribution to the work, and approved it for publication.

## Conflict of Interest

The authors declare that the research was conducted in the absence of any commercial or financial relationships that could be construed as a potential conflict of interest.

## Footnotes

1 http://www.genomatix.ed

2 https://david.ncifcrf.gov/tools.jsp

3 http://www.targetscan.org/

4 http://www.microrna.org/

5 https://pictar.mdc-berlin.de/

6 https://genie.weizmann.ac.il/

7 https://cm.jefferson.edu/rna22/
